# CAUSAL EFFECTS OF SCHOOLING ON MEMORY AT OLDER AGES IN FIVE LOW- AND-MIDDLE-INCOME COUNTRIES

**DOI:** 10.1093/geroni/igae098.0278

**Published:** 2024-12-31

**Authors:** Shana Stites, Vikesh Amin, Jere Behrman, Jason Fletcher, Carlos Flores, Alfonso Flores-Lagunes, Iliana Kohler, Hans-Peter Kohler

**Affiliations:** University of Pennsylvania, Philadelphia, Pennsylvania, United States; Central Michigan University, Mount Pleasant, Michigan, United States; University of Pennsylvania, Philadelphia, Pennsylvania, United States; University of Wisconsin Madison, Madison, Wisconsin, United States; California Polytechnic State University, San Luis Obispo, California, United States; Syracuse University, Syracuse, New York, United States; University of Pennsylvania, Philadelphia, Pennsylvania, United States; University of Pennsylvania, Philadelphia, Pennsylvania, United States

## Abstract

We investigate the relationships between early life schooling and performances on memory tests at older ages in China, Ghana, India, Mexico, and South Africa using harmonized data from the World Health Organization Study on Global Ageing and Adult Health. We apply a nonparametric partial-identification approach that bounds causal effects of increasing schooling at different parts of the schooling distributions under relatively weak assumptions. We find that an extra grade of schooling is associated with memory scores that are higher by at most 0.12 standard deviations (SDs) in China, 0.06 SDs in Ghana, 0.10 SDs in India, 0.09 SDs in Mexico, and 0.07 SDs in South Africa, between never-attending school and completing primary schooling. The bounds statistically rule out null effects for all countries except South Africa. At higher parts of the schooling distributions (e.g., high-school or university completion) the bounds cannot statistically rule out null effects.

